# Test-time augmentation with synthetic data addresses distribution shifts in spectral imaging

**DOI:** 10.1007/s11548-024-03085-3

**Published:** 2024-03-14

**Authors:** Ahmad Bin Qasim, Alessandro Motta, Alexander Studier-Fischer, Jan Sellner, Leonardo Ayala, Marco Hübner, Marc Bressan, Berkin Özdemir, Karl Friedrich Kowalewski, Felix Nickel, Silvia Seidlitz, Lena Maier-Hein

**Affiliations:** 1 Division of Intelligent Medical Systems (IMSY), German Cancer Research Center (DKFZ), Heidelberg, Germany; 2 Helmholtz Information and Data Science School for Health, Karlsruhe/Heidelberg, Germany; 3 Faculty of Mathematics and Computer Science, Heidelberg University, Heidelberg, Germany; 4 Department of General, Visceral, and Transplantation Surgery, Heidelberg University Hospital, Heidelberg, Germany; 5 National Center for Tumor Diseases (NCT), NCT Heidelberg, A Partnership between DKFZ and University Medical Center Heidelberg, Heidelberg, Germany; 6 Department of Urology, University Medical Center Mannheim, Heidelberg University, Mannheim, Germany; 7 Medical Faculty, Heidelberg University, Heidelberg, Germany

**Keywords:** Hyperspectral imaging, Deep learning, Surgical scene segmentation, Tissue classification, Domain generalization, Test-time augmentation

## Abstract

**Purpose:**

Surgical scene segmentation is crucial for providing context-aware surgical assistance. Recent studies highlight the significant advantages of hyperspectral imaging (HSI) over traditional RGB data in enhancing segmentation performance. Nevertheless, the current hyperspectral imaging (HSI) datasets remain limited and do not capture the full range of tissue variations encountered clinically.

**Methods:**

Based on a total of 615 hyperspectral images from a total of 16 pigs, featuring porcine organs in different perfusion states, we carry out an exploration of distribution shifts in spectral imaging caused by perfusion alterations. We further introduce a novel strategy to mitigate such distribution shifts, utilizing synthetic data for test-time augmentation.

**Results:**

The effect of perfusion changes on state-of-the-art (SOA) segmentation networks depended on the organ and the specific perfusion alteration induced. In the case of the kidney, we observed a performance decline of up to 93% when applying a state-of-the-art (SOA) network under ischemic conditions. Our method improved on the state-of-the-art (SOA) by up to 4.6 times.

**Conclusion:**

Given its potential wide-ranging relevance to diverse pathologies, our approach may serve as a pivotal tool to enhance neural network generalization within the realm of spectral imaging.

## Introduction

Semantic segmentation of intraoperative imaging data plays a crucial role in context-awareness and autonomous robotics in surgery. Spectral imaging [1] has emerged as an alternative to RGB imaging for intraoperative use, because it offers entirely new possibilities for recovering functional and morphological information. Examples include perfusion monitoring [2–5], tumor detection [6–8] and tissue differentiation [9–13]. Unlike RGB imaging, which imitates human perception and is based on solely three channels in the visible spectrum of light, it is based on an arbitrary number of channels across a potentially wider spectral range. The term multispectral imaging (MSI) is commonly used for spectral imaging with up to tens of spectral bands, while spectral imaging with up to hundreds of spectral bands is termed hyperspectral imaging (HSI) [1].

**Fig. 1:**
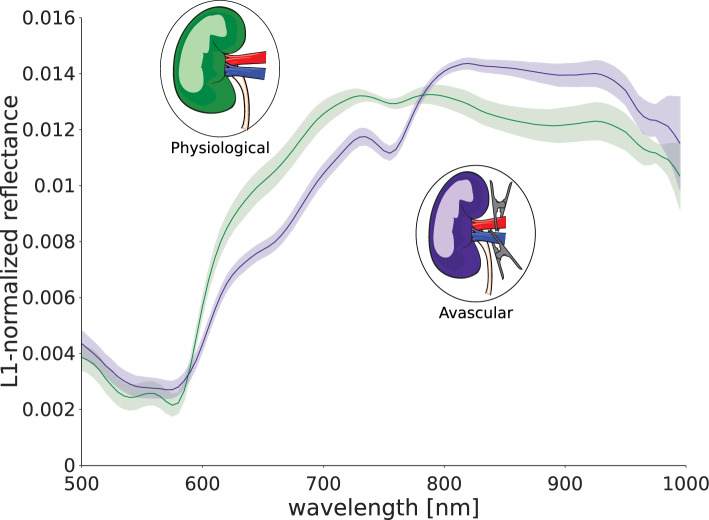
Surgical intervention as well as pathologies can lead to extreme domain shifts between training and deployment datasets. This holds true especially for new imaging modalities (here: hyperspectral imaging (HSI)) for which training data is sparse. In this specific scenario, training data for a surgical scene segmentation algorithm were acquired from well-perfused organs (green) and does not represent poorly perfused tissue (purple) well

Recent advances in deep learning-based surgical scene segmentation using HSI have achieved performances on par with human expertise [13]. However, this research has largely been based on subjects without previous surgical alterations or pre-existing health conditions. This limitation, which can be attributed to a lack of data adequately capturing the diverse spectrum of tissue variations encountered in clinical settings, substantially impedes the generalization of the developed machine learning (ML) models. Addressing this gap in the literature, this work investigates the impact of perfusion variations resulting from surgical interventions on the tissue discrimination performance of state-of-the-art (SOA) ML models. As depicted in Fig. 1, these perfusion-induced variations can give rise to a challenging distribution gap between data representing physiological and pathological conditions, potentially hindering the generalization capabilities of ML models to such scenarios. The contribution of this paper is twofold. Firstly, we demonstrate that distribution shifts resulting from perfusion changes can lead to a dramatic decline in the performance of HSI-based tissue classification algorithms. Secondly, we introduce a novel test-time augmentation approach that leverages synthetic HSI data to overcome perfusion-related distribution shifts.

## Materials and methods

Our methodology is grounded in the following critical observations.

*Sparsity of real-world data:* training data from emerging imaging modalities, such as HSI, lacks the diversity to represent the full range of pathologies encountered in real-word medical settings. For example, the largest publicly available HSI data set in visceral surgery, HeiPorSpectral [14], solely features images from well-perfused tissue.

*Limitations of synthetic data generation:* the generation of synthetic HSI data is challenging. While a lot of progress has been made in simulating plausible HSI spectra [15], we are not aware of any prior work on synthesizing full hyperspectral surgical scenes. Furthermore, the challenge of conditioning synthetic spectra generation on one of many tissue classes has not yet been addressed. This would be an important prerequisite for training semantic scene segmentation methods based on HSI data with pixel-wise class labels.

**Fig. 2:**
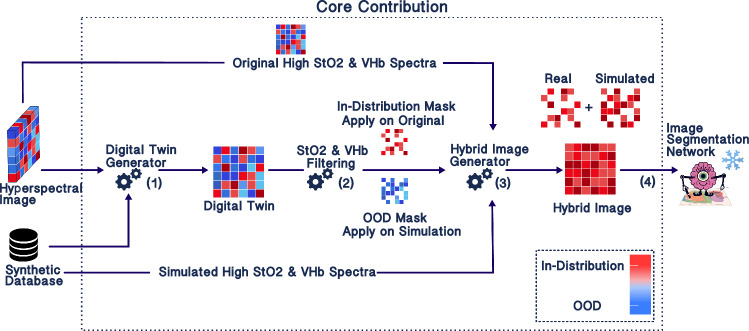
Test-time augmentation for addressing perfusion-related domain shifts in the context of surgical scene segmentation. (1) The hyperspectral image is converted into its synthetic digital twin using a synthetic database of plausible tissue geometries with corresponding spectra and functional tissue parameters such as oxygen saturation (StO2) and blood volume fraction (VHb). (2) The pixels with OOD tissue perfusion are identified and (3) augmented based on in-distribution synthetic spectra. (4) This yields a hybrid hyperspectral image comprising both original spectra and augmented spectra, which is processed by a frozen model to perform semantic scene segmentation

In response to these shortcomings, our approach takes the form of a “best-of-both-worlds” strategy, tailored to address perfusion shifts in HSI analysis with the help of both real and synthetic data. We assume that the real data comprising full HSI images with pixel-wise class labels represents only a limited number of perfusion conditions that can be encountered in practice (e.g., only physiological in Fig. 5). The synthetic data, on the other hand, lack the tissue labels and global context but represent a broader range of perfusion conditions. This is achieved by configuring the spectrum generation pipeline in Sect. 2.2.3 with extreme (even implausible) parameter values for oxygen saturation (StO2) (0–100%) and blood volume fraction (VHb) (0–30%). Inspired by the concept of test-time augmentation, our method combines these two data sources to transform real-world images from unseen perfusion states into input images that align with the training data distribution. This enables the application of a frozen SOA network without retraining, as depicted in Fig. 2.

The subsequent sections provide an overview of the proposed concept which is given in Sect. 2.1, the datasets employed in this study which are presented in Sect. 2.2, a preliminary prototype implementation of the approach which is given in Sect. 2.3, and details of the experimental conditions which are presented in Sect. 2.4.

### Concept overview

While the general idea of this paper is in principle applicable to a broad range of pathologies, the study presented here has specifically been designed for perfusion state shifts in hyperspectral image classification. Our assumption is that a neural network for organ segmentation has been trained on well-perfused (potentially healthy) tissue, as in previous studies [9, 13], and is then applied to real-world settings in Fig. 1. The basic idea to address perfusion-related distribution shifts is illustrated in Fig. 2. The foundation of our pipeline is a synthetic tissue database comprising a large volume of plausible tissue spectra, generated with the help of a device digital twin of the HSI camera used for our study (see Sect. 2.2.3). Test-time augmentation is achieved in three steps:

- Step 1—Digital twin generation: initially, the input HSI image is converted to its corresponding tissue digital twin using the synthetic tissue database. This yields a hyperspectral image annotated with relevant tissue parameters including corresponding StO2 and VHb.
- Step 2—StO2 and VHb filtering: next, the tissue parameters are leveraged to identify pixels corresponding to out-of-distribution (OOD) perfusion states. OOD perfusion states, refers to states which were not present during training of the segmentation network.
- Step 3—Hybrid image generation: the synthetic tissue database is leveraged to convert OOD pixels to in-distribution pixels. The final test-time-augmented image is composed of the original in-distribution pixels and pixels transformed based on the synthetic database and can then be fed into the frozen segmentation network.

A concrete implementation of this concept is provided in Sect. 2.3.

### Hyperspectral imaging data

The data used for the development and validation of our approach comprise 511 hyperspectral images from 12 porcine models in which the perfusion of the kidney was altered (Sect. 2.2.1), 104 hyperspectral images from 4 porcine models in which the perfusion of several abdominal organs was altered through clamping of the aorta (Sect. 2.2.2) as well as 500,000 synthetic tissue spectra simulated with a Monte Carlo-based approach (Sect. 2.2.3). For real-world data acquisition, the TIVITA$\circledR$ Tissue Halogen system (Diaspective Vision GmbH, Am Salzhaff, Germany) was used. This system illuminates the respective field of view of around 20 $\times$ 27 cm with six integrated halogen lamps and provides a spectral resolution of 5 nm in the range from 500nm to 995nm for every recorded pixel. The synthetic data were generated with a digital twin of the same camera.

#### In vivo porcine kidney data

We covered the following perfusion scenarios on 12 pigs undergoing kidney surgery:

1. *Physiological* tissue: we acquired 213 images without altering kidney arterial inflow or venous outflow. These images are in-distribution with the training data.
2. *Avascular* tissue: on 108 images, both arterial inflow and venous outflow were inhibited through reversible clamping. This scenario emulates a transplantation procedure.
3. *Arterial ischemia*: the arterial inflow to the kidney was inhibited while venous outflow was not restricted for a total of 113 images. This scenario emulates for example a partial nephrectomy procedure, in which arteries of the kidney are clamped to resect a tumor.
4. *Venous congestion*: we acquired 77 images with inhibited kidney venous outflow and unrestricted arterial inflow, akin to conditions like venous thrombosis or a blocked anastomosis of the vein during transplantation.

In the OOD scenarios, the clamping was repeated several times for a clamping period of at least 2 mins. Hyperspectral images were taken every 30 sec throughout the clamping period. The slight heterogeneity in the number of acquired images results from the exclusion of images with uncertain perfusion state. Reference kidney annotations were generated by a medical expert using a polygon tool.

#### In vivo porcine aorta clamping data

We covered the following perfusion scenarios on 4 pigs undergoing aorta clamping during surgery:

1. *Aortic ischemia*: we acquired 80 hyperspectral images during and after blocking the blood flow of the aorta using a removable clamp. As a consequence of the supradiaphragmatic aortic clamping, a variety of visceral organs, including the colon, small bowel, and liver, are expected to become ischemic. This scenario emulates a range of clinical scenarios, including systemic malperfusion states as a consequence of, e.g., cardiac insufficiency, as well as organ-specific ischemia occurring as a side effect in general surgeries (e.g., oncological resections). This scenario allows us to simultaneously observe the effect of arterial ischemia on the colon, small bowel, and liver.
2. *Reperfusion*: after 20 min of aortic clamping, the clamp was removed, leading to the reperfusion of the organs. During the first 6 min of reperfusion, 24 images were acquired. Hyperspectral images were generally taken every 1 min during the aortic ischemia and reperfusion.

Upon initial sedation, the animals were intubated and anesthetized. Body temperature, peripheral oxygen saturation, blood gas analysis parameters and the flow through the renal artery were monitored throughout the measurements to rule out potential confounding factors.

#### Synthetic data

The synthetic data in our study were generated through the simulation of light transport within a generic tissue model using a Monte Carlo method and based on a range of parameters relevant to the image formation process, including StO2 and VHb [16]. More specifically, the synthetic dataset comprises 500,000 reflectance spectra ranging from 300 nm to 1000 nm that were generated according to [15] using a 2-nm spacing between the wavelengths and 106 photons per wavelength. The underlying physiological tissue model has three tissue layers. The ranges of the optical properties characterizing the tissue model were derived from the literature [4], namely StO2 (0–100%), VhB (0–30%), reduced scattering coefficient (5–5 cm^-1^), scattering power [0.3–3 arbitrary units (a.u.)], anisotropy (0.8–0.95 a.u.), refractive index (1.33$-$1.54 a.u.), tissue thickness (0.002–0.2 cm) and water content (0.8–0.9 a.u.). The simulations have been conducted with a GPU-accelerated version [17] of the Monte Carlo multilayered simulation framework [18].

### Prototype implementation of test-time augmentation

The following paragraphs describe implementation details of the first prototype implementation of the proposed test-time augmentation approach. *The digital twin generator* operates through a pixel-wise nearest neighbor search within the synthetic database. This process allows us to retrieve the simulation parameters while maintaining a spectrum that closely resembles the original. *The detection of OOD* pixels relies on the prior knowledge acquired during network training on physiological data. The data set used has been described in previous work [13] and comprises 506 HSI images from 20 pigs with 18 different tissue types, namely heart, lung, stomach, small intestine, colon, liver, gallbladder, pancreas, kidney, kidney with Gerota’s fascia, spleen, bladder, subcutaneous fat, skin, muscle, omentum, peritoneum and major veins. To detect OOD pixels, we check whether the StO2 and VHb values of the corresponding digital twin pixels lie within the interquartile range (IQR) of StO2 and VHb values for the physiological training data. Note in this context that we are—strictly mathematically speaking—not checking whether a given real pixel is in the distribution of the training data (which is itself subject of ongoing research). Instead we make the OOD decision based on properties that we can directly correct for (StO2 and VHb).

*Hybrid image generator:* pixels that are considered to be in-distribution undergo no changes. OOD pixels are corrected by finding the nearest neighbor in the synthetic database that features StO2 and VHb values close to the median StO2 and median VHb observed in the physiological data. To compensate for the fact that purely synthetic data may not be fully realistic, we then average the real spectrum with the synthetic one to obtain the final transformed spectrum. The resulting hybrid image is inputted into a pre-trained frozen network.

*Segmentation network:* segmentation performance on par with human inter-rater variability was achieved by Seidlitz et al. [13] using a U-Net architecture with an efficientnet-b5 encoder. The related network training was recently improved to yield better generalization across geometric domain shifts [9] and the corresponding network weights were made publicly available at https://github.com/IMSY-DKFZ/htc. This pre-trained segmentation network is used here without any further fine-tuning. As input, it takes full hyperspectral images with L1-normalized spectra.

### Experiments

The purpose of the experiments was to investigate the following two research questions:

RQ1: Do abnormal perfusion states lead to domain shifts that cause SOA surgical scene segmentation algorithms to fail?

RQ2: Can test-time augmentation with synthetic data compensate for perfusion-related domain shifts?

To this end, we validated our prototype implementation using the real-world datasets described in Sects. 2.2.1 and 2.2.2.

To qualitatively assess the domain shift between physiological and malperfused kidneys, median kidney spectra per image were computed for all 511 images in the dataset before and after test-time augmentation. Principal component analysis (PCA) was then performed on the union of all original and augmented median spectra from all four perfusion states. Kernel density estimation (KDE) was used to approximate the probability density function of the spectra for each perfusion state. The differences between the density functions before and after test-time augmentation were visually compared. Furthermore, the change in the median spectra was visually compared.

To quantitatively assess the effect of perfusion-induced domain shifts on segmentation performance, segmentation predictions were generated for the original and augmented images using the frozen SOA network described in Sect. 2.3. The widely used Dice similarity coefficient (DSC) [19] was used to compare the predictions to reference semantic annotations for the kidney (cf. Sect. 2.2.1) as well as colon, small bowel, and liver (cf. Sect. 2.2.2). The DSC scores for each individual image and organ class were hierarchically aggregated to derive overall organ scores.

To avoid model overfitting, we randomly split the in vivo kidney data comprising 511 images from 12 pigs into a validation set comprising 341 images from 7 pigs and a hold-out test set comprising 170 images from 5 pigs. On the validation set, two different thresholds for the OOD detection were used: a more conservative setting defining the range 5–95 percentile as in-distribution and a more comprehensive setting defining only the range 25–75 as in-distribution. A decision was made based on the overall DSC on the validation data. After setting the thresholds, the DSC performance on the test set was analyzed. The aorta clamping data (cf. Sect. 2.2.2) were treated as an additional test set.

**Fig. 3:**
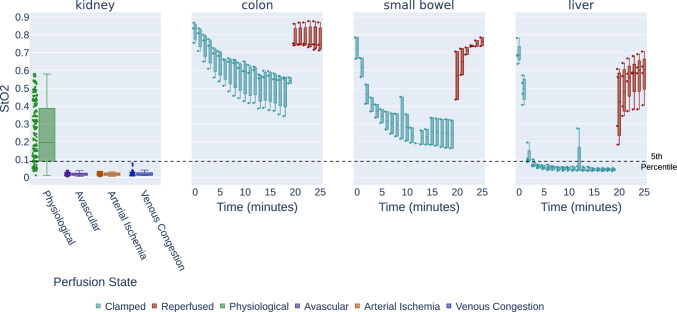
StO2 changes resulting from tissue manipulation according to synthetic digital twin analysis. Each box plot depicts the time-resolved mean StO2, retrieved from the nearest neighbor simulated spectrum and averaged over all images and subjects. The black dotted line on the 0.09 represents the OOD threshold of StO2 that we used in this study

**Fig. 4:**
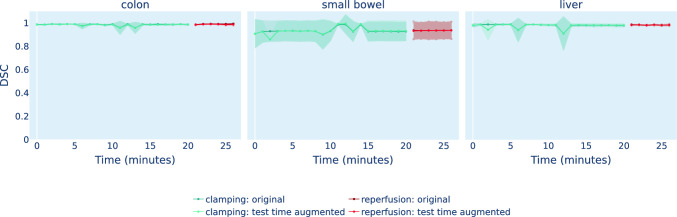
Aorta clamping does not lead to a segmentation performance drop. For each organ, the line plot presents the time-resolved DSC hierarchically averaged for each subject over each timepoint. The first 20 mins depict clamping, while the last 6 mins depict reperfusion. The standard deviation over subjects is shown as the shaded area around the line plot

**Fig. 5:**
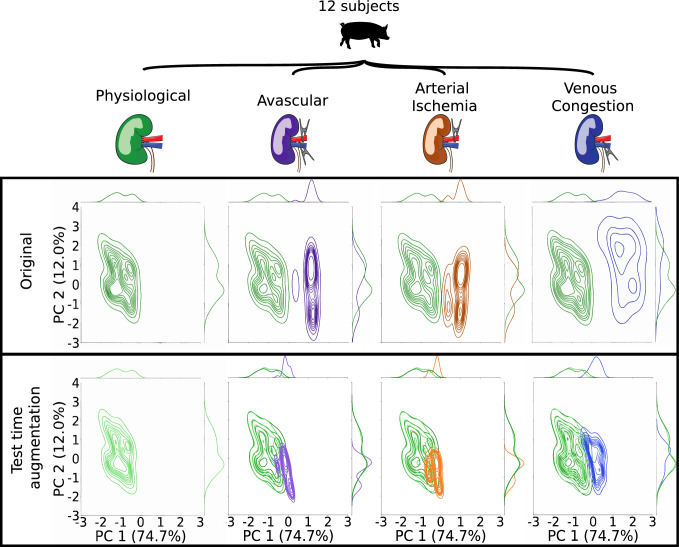
Our test-time augmentation approach reduces the domain gap between training and deployment data. For each perfusion condition (physiological, avascular, arterial ischemia and venous congestion) the kernel density estimation of the median spectrum per image is depicted before (upper row) and after (lower row) test-time augmentation. After augmentation, the density of the physiological data (representing the training data) is in much better agreement with the deployment data. For illustration, the dimensionality of the data was reduced by a principal component analysis (PCA). The variance captured by the first and second principal axis was 74.7% and 12.0%, respectively, across all 511 original and 511 augmented median spectra obtained from 12 subjects

**Fig. 6:**
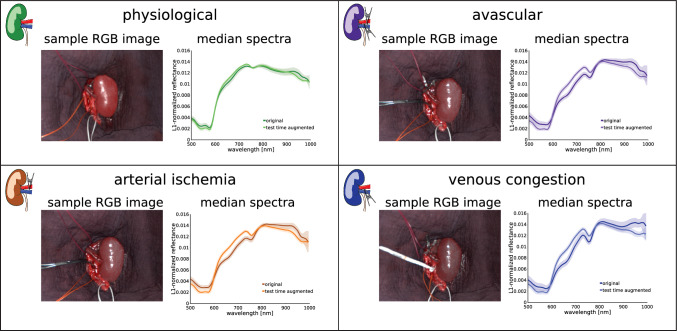
Representative images and spectra for the perfusion conditions **a** physiological, **b** avascular, **c** arterial ischemia and **d** venous congestion. For each state, the median spectrum across all 5 test pigs before and after test-time augmentation is depicted

## Results

RQ1: Do abnormal perfusion states lead to deep learning failure?

**Fig. 7:**
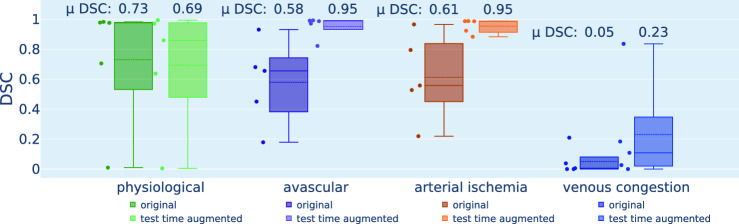
Our test-time augmentation approach improves kidney segmentation under perfusion shifts. The box plots present the Dice similarity coefficient (DSC) hierarchically averaged for each subject. Median and mean values are shown as solid and dashed lines, respectively. The boxes represent the interquartile range (IQR) and whiskers extend up to 1.5 times the IQR

While a drop in StO2 during the aortic clamping period and a recurrence of physiological StO2 levels upon reperfusion can be observed for the organ classes colon, small bowel, and liver (cf. Fig. 3), the segmentation network performance did not deteriorate for any of the three organ classes throughout the entire time course (cf. Fig. 4). Instead, the DSC remains consistently close to 1 for all recordings. However, in the case of kidney, Fig. 5 demonstrates a large domain gap between well-perfused and poorly perfused tissue. Representative spectra are depicted in Fig. 6. This gap leads to a failure of organ segmentation algorithms. In fact, the DSC for the kidney drops from 0.73 (physiological), to 0.58 (avascular), 0.61 (arterial ischemia) and 0.05 (venous congestion), respectively, which corresponds to a relative decrease in performance of up to 93% as shown in Fig. 7.

*RQ2: Can test-time augmentation compensate for the effect?*

According to Figs. 5 and 6, our test-time augmentation approach substantially reduces the domain gap between training and deployment data. This has a direct positive effect on the downstream task performance, as illustrated in Fig. 7. Compared to the baseline approach (no augmentation), our method improves the DSC by 0.37 (avascular), 0.34 (arterial ischemia) and 0.18 (venous congestion), respectively. This corresponds to relative improvements by factors of 1.63, 1.55 and 4.6, respectively. In the aortic clamping scenario, in which a performance drop with perfusion alterations could not be observed, the network performance with and without test-time-augmentation was on par (cf. Fig. 4).

## Discussion

To our knowledge, this paper is the first to study the effect of perfusion shifts on the performance of HSI segmentation algorithms. We showed that perfusion conditions encountered in real-world settings but not during neural network training can have a devastating effect on a model’s tissue classification performance. To overcome this issue, we proposed a test-time augmentation approach, with which we were able to move the test data distribution closer to the training data distribution and therewith substantially enhance the performance.

With this work, we address a key gap in the literature. Previous work on surgical scene segmentation has focused on data shifts related to geometry [9], but we are not aware of any publications that address the lack of abnormal conditions or specific pathologies in the training data. In different domains, other approaches such as disentangled representation learning [20], fine-tuning [21, 22] and style transfer [23, 24] have been used to overcome certain types of domain shifts. While such approaches have shown promise in other domains, they are mostly not applicable to our problem and typically rely on the availability of large amounts of data, often thousands of images. The latter ultimately renders such approaches unusable under the conditions of data scarcity typical for the surgical domain, especially when dealing with novel image modalities such as HSI. To overcome this gap in the literature, we propose a different approach that encapsulates valuable prior knowledge about the origin of the domain shift.

In this context, we are unaware of any prior work in the broader field of surgical scene understanding that has utilized test-time augmentation.

Outside the field of surgical scene segmentation, several approaches for test-time augmentation have been proposed in the broader context of image classification and semantic segmentation in non-medical settings. One approach [25–28] is to modify the training paradigm by changing the network architecture, such that the architecture can be adapted to the test set distribution on-the-fly. Such training paradigms are termed test time training paradigms. However, such paradigms require the model to be retrained using the new architecture and thus require access to the original training data. Furthermore, often in medical applications, accurately determining the test set statistics is not possible in an online setting, as the complete test set is not available for inference at a given time. The same challenge is faced by the approaches [29–32], which involve changing or fine-tuning the batch normalization [33] statistics of the trained model, to match the statistics of the complete test set. Additionally, it has been shown that adapting batch normalization statistics is not sufficient for more challenging tasks [34–36]. Batch-agnostic normalization layers (e.g., group normalization [37]) have been shown to be more robust toward more challenging tasks. However, they still depict failure cases [36]. Meanwhile, although conditional autoencoder, GANs [38] and diffusion models [39] have been shown to be able to transfer the test distribution to the original training distribution for test time adaptation [40–42], these models require a large amount of data to generalize well to unseen domains. On the other hand, the medical HSI field is very limited in terms of availability of large datasets.

A key strength of our method is that it does not require retraining the network; instead, the incoming data are transferred so that it can be handled by a *frozen* network. Furthermore, it elegantly leverages *prior knowledge* on potential gaps between training and deployment datasets. While, to correct for OOD pixels, our method increases inference time, we assume that it can be optimized to be real-time capable.

Overall, our work represents a promising, novel concept, but several limitations and opportunities for future work deserve further discussion:

Furthermore, while we obtained a performance boost of up to a factor of 4.6, performance for venous congestion is not on par with in distribution performance. This can possibly be explained with the shortcomings of our simulation framework. For example, our simulations only consider the most obvious consequences of perfusion shifts, namely changes in blood volume fraction (VHb) and oxygen saturation (StO2). However, venous congestion, for example, can lead to the accumulation of several substances in the kidney (e.g., azotemia), as the kidney plays a major role in filtering waste products from the body. Changes in the concentration of chromophores other than Hb/HbO2 have not been considered in the simulations, but may alter the spectra and thus lead to poor baseline performance in case of venous congestion. Overall, integration of pathologies in simulation frameworks such as [43] is an open research topic.

Our study addresses domain shifts related to perfusion, specifically exploring clinical scenarios that involve entire organs affected by arterial ischemia, venous congestion, or avascular conditions. While we consider the data acquired for this study to be unique, future work should expand the application of our approach to a wider range of perfusion states and pathologies. This expansion should include validation on common surgical scenarios such as partial perfusion impairment (e.g., reduced inflow or outflow, only parts of an organ being affected by malperfusion). A key remaining research question in this context is how to transfer the proposed approach to further conditions, such as cancerous, cirrhogenous or fatty tissue. Multiple works have investigated the capabilities of HSI to discriminate physiological and pathological tissues, indicating that their spectral signatures can be very distinct [44]. We therefore assume that, equivalent to our findings for perfusion state shifts, domain gaps between physiological and pathological tissue could deteriorate the performance of a segmentation network that was solely trained on physiological data. As is the case for perfusion-induced variations, real-world pathological HSI data is very sparse with only a single publicly available dataset that is small and covers only a few specific brain tumors [45]. While synthetic data generation to simulate perfusion variations is established, the simulation of pathological tissue alterations has not yet been addressed due to a lack of substantial prior knowledge (e.g., measurements of optical properties for pathological tissues). Closing this knowledge gap is an important next step to enable the transfer of our approach to pathology-induced domain gaps.

With regard to our study’s design, it could also be argued that we should have based our study on a simple pixel-wise classification network. However, previous work [9] showed that the model performance increases with increased spatial granularity of HSI data. As a simple pixel-wise classification network did not yield performance comparable to human experts, we based our study on image-wise segmentation. Furthermore, phrasing it as a segmentation task enabled us to base our work on an openly available network, thus enabling the comparison of performance values.

Finally, our prototype implementation comes with a relatively simple way to detect and replace OOD pixels. More sophisticated methods can potentially further boost performance. It should be noted that we avoided hyperparameters in our method due to the limited number of validation cases. Had we had access to more data, for example, we could have tuned the threshold for deciding whether a sample is OOD.

In conclusion, this paper pioneered the exploration of distribution shifts in spectral imaging caused by perfusion alterations. Our test-time augmentation-based approach could evolve as a blueprint for addressing further domain shifts resulting from surgical intervention or pathologies.
